# Supplementary material for: Predicting Spin-Dependent Phonon Band Structures of HKUST-1 Using Density Functional Theory and Machine-Learned Interatomic Potentials
Source: Int J Mol Sci. 2024 Mar 5;25(5):3023. doi: 10.3390/ijms25053023 (PMC10931957; doi:10.3390/ijms25053023)
Supplement: Supplementary file 1 [file ijms-25-03023-s001.zip › supplementary materials.pdf]

International Journal of *Molecular Sciences*

MDPI

Supplementary Materials

Predicting Spin-Dependent Phonon Band Structures of HKUST-1 Using Density Functional Theory and Machine-Learned Interatomic Potentials

Nina Strasser, Sandro Wieser and Egbert Zojer *

Institute of Solid State Physics, NAWI Graz, Graz University of Technology, 8010 Graz, Austria; nina.strasser@tugraz.at (N.S.); sandro.wieser@alumni.tugraz.at (S.W.)

* Correspondence: egbert.zojer@tugraz.at

Table of Contents

S1. Geometry parameters of HKUST-1 with and without spin polarization 2

S2. Spin-dependent vibrations of HKUST-1 in the low-frequency regime 3

S3. Comparison of DFT/PBE- and MTP-calculated phonon band structures in the intermediate-frequency range 4

S4. Eigenvector overlaps of Γ-point phonons between DFT and machine-learned force fields for HKUST-1 5

S5. Convergence tests on the total energy regarding the plane-wave cutoff and the k-grid 11

S6. Convergence of the supercell size of HKUST-1 for phonon calculations using machine-learned force fields 12

S7. Performance of the MTPs 15

S8. Comparison of PBE-derived phonon frequencies calculated with VASP and FHI-aims including the impact of the van der Waals correction 16

S9. Impact of using a hybrid functional on phonon frequencies 21

S10. Comparison between simulated and experimental IR data 25

S1. Geometry parameters of HKUST-1 with and without spin polarization

This section reports the results of the cell lengths (a, b, c), angles (α, β, γ), and unit cell volumes (V) as a function of the spin configuration for the relaxed trigonal primitive unit cell of HKUST-1 using DFT/PBE in Table S1. No symmetry constraints were enforced during the relaxation of the structure in VASP, allowing for a variation of cell lengths and atomic positions. The NM configuration has the smallest unit cell, followed by the AFM state, while the FM state has the most expanded unit cell, which is also reflected in the lattice cell lengths and cell volumes.

Table S1. Geometric parameters describing the primitive unit cells of HKUST-1 in the NM, FM and AFM spin states calculated employing the PBE functional and the D3 van der Waals correction. The calculated lengths of the unit cell vectors (a, b and c), the angles between them (α, β and γ) and the cell volumes (V) are given. The average nearest neighbor Cu-Cu, Cu-O and C=C bond lengths within the benzene rings are reported as well.

	**a [Å]**	**b [Å]**	**c [Å]**	**α [°]**	**β [°]**	**γ [°]**	**V [Å^3]**	**Cu-Cu [Å]**	**Cu-O [Å]**	**C=C [Å]**
**AFM**	18.736	18.725	18.733	60.03	59.99	59.98	4647	2.457	1.966	1.399
**FM**	18.744	18.744	18.745	60.00	60.00	60.00	4656	2.440	1.968	1.399
**NM**	18.708	18.709	18.709	60.00	60.00	60.00	4630	2.480	1.967	1.399

In order to assess, whether structural variations of HKUST-1 between the NM, FM and AFM states could be identified by diffraction experiments, diffractograms were calculated using Mercury [84]. They are shown in Figure S1. As already mentioned in the Results and Discussion section in the main manuscript, there is no noticeable difference between the diffractograms for the different states of HKUST-1.

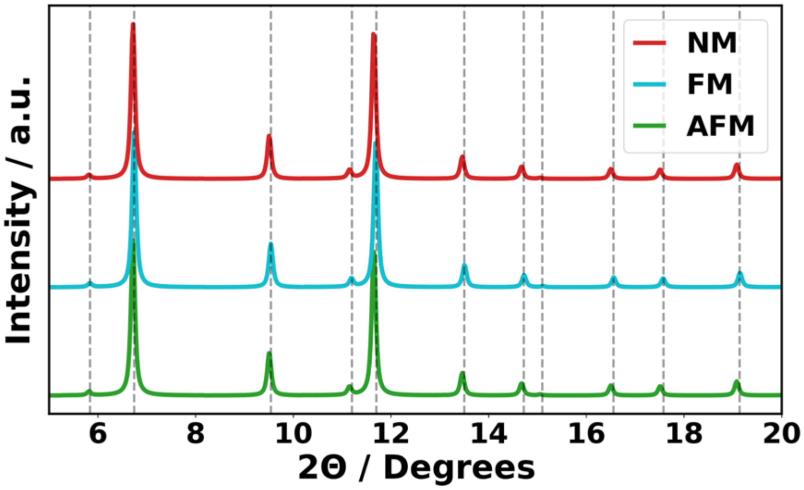

Figure S1. Diffraction patterns of HKUST-1 in the NM, FM and AFM state computed with VASP using the PBE functional in the range from 5 degrees to 20 degrees using a wave length of 1.54 Å (Cu-K$_\alpha$ radiation).

S2. Spin-dependent vibrations of HKUST-1 in the low-frequency regime

As discussed in the Results section of the main paper in the context of Figure 3, we observe that the frequencies of certain optical phonons of HKUST-1 in the low-frequency region depend on the spin state of the material. A comprehensive compilation of vibrations for the AFM, FM and NM state along with the names of the files containing animations of representative vibrations, is provided in Table S2 where similar types of vibrations are grouped and separated by horizontal lines. The animations were generated using OVITO (version: 3.10) [85].

Table S2. Low-frequency vibrations for HKUST-1 in the AFM, FM and NM state calculated using DFT with the PBE functional. The names of animations for the vibrations in the primitive trigonal unit cell of HKUST-1 for the AFM state are included.

AFM [THz]	FM [THz]	NM [THz]	Name of Animation File
0.562<br>0.563<br>0.565	0.540<br>0.541<br>0.541	0.506<br>0.521<br>0.522	HKUST-1_0.562THz.mp4
0.858<br>0.858	0.880<br>0.880	0.717<br>0.717	HKUST-1_0.858THz.mp4
1.225<br>1.231<br>1.234	1.161<br>1.162<br>1.163	1.082<br>1.084<br>1.084	HKUST-1_1.225THz.mp4
1.338<br>1.340<br>1.354<br>1.364<br>1.379<br>1.392	1.334<br>1.345<br>1.346<br>1.347<br>1.386<br>1.386	1.093<br>1.150<br>1.150<br>1.289<br>1.292<br>1.294	HKUST-1_1.338THz.mp4
1.848<br>1.855<br>1.860<br>1.903<br>1.904<br>1.905	1.697<br>1.698<br>1.700<br>1.988<br>1.988<br>1.989	1.599<br>1.605<br>1.610<br>1.988<br>1.989<br>1.993	HKUST-1_1.848THz.mp4
2.113<br>2.115<br>2.116	2.083<br>2.086<br>2.087	2.083<br>2.086<br>2.087	HKUST-1_2.113THz.mp4

2.438	2.384	2.382	HKUST-1_2.438THz.mp4
2.583	2.449	2.448	HKUST-1_2.583THz.mp4
2.727<br>2.767<br>2.769<br>2.770	2.527<br>2.591<br>2.592<br>2.595	2.527<br>2.591<br>2.592<br>2.595	HKUST-1_2.727THz.mp4

S3. Comparison of DFT/PBE- and MTP-calculated phonon band structures in the intermediate-frequency range

The phonon band-structures in the low-frequency region calculated with DFT/PBE and the MTPs of HKUST-1 have already been compared in Figure 8. Figure S2 contains an equivalent comparison for the frequency region between 38 THz and 50 THz, which is most affected by variations in the spin conformation. The majority the optical bands in this region are rather flat and the MTPs reproduce the phonon dispersion reasonably well. Still, the discrepancies of the positions of the Γ-point frequencies (already discussed in the main manuscript) result in DFT/PBE and MTP bands being shifted relative to each other. Moreover, certain bandwidths are over-, respectively, underestimated by the MTPs. This is illustrated by a zoom into the region from 39 THz to 42 THz in Figure S3. The only larger deviation is observed for the bands located at around 46.3 THz in the AFM case, as already discussed in the main paper. These bands correspond to -COO- stretching modes.

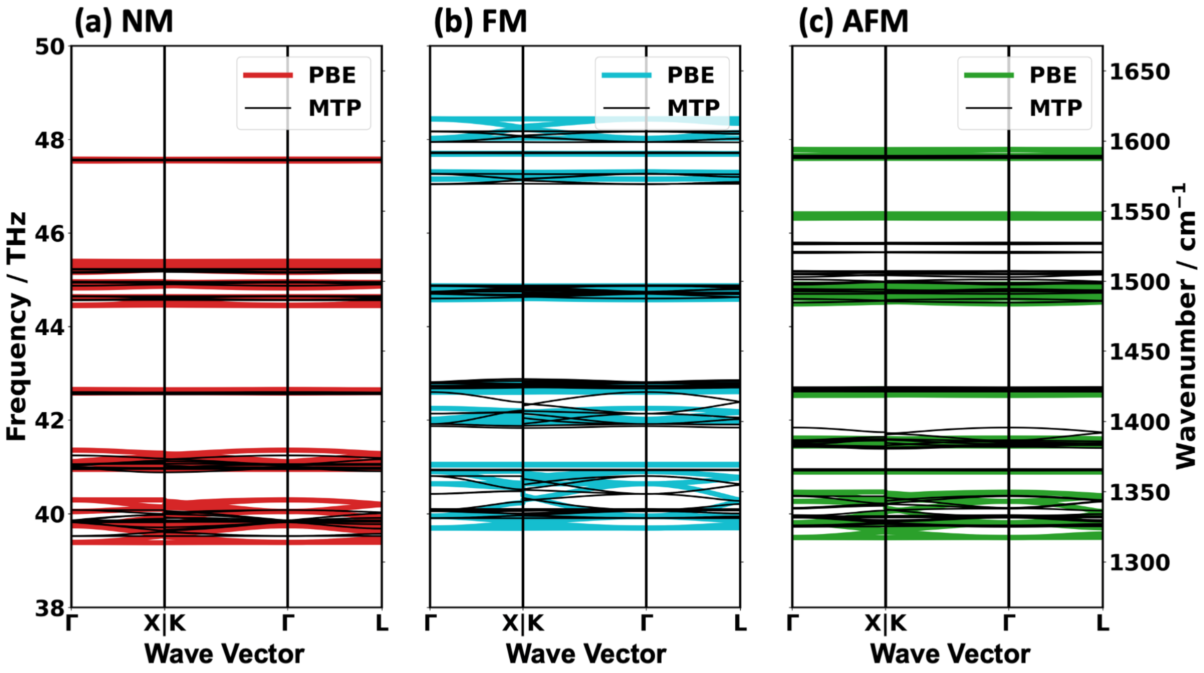

Figure S2. Phonon band structure comparison between the phonon modes in the frequency region from 38 THz to 50 THz of HKUST-1 in the NM (**a**), FM (**b**) and AFM states (**c**). The DFT/PBE results are shown with thick colored lines and the predictions made by the spin-dependent MTPs are added using thinner black lines on top.

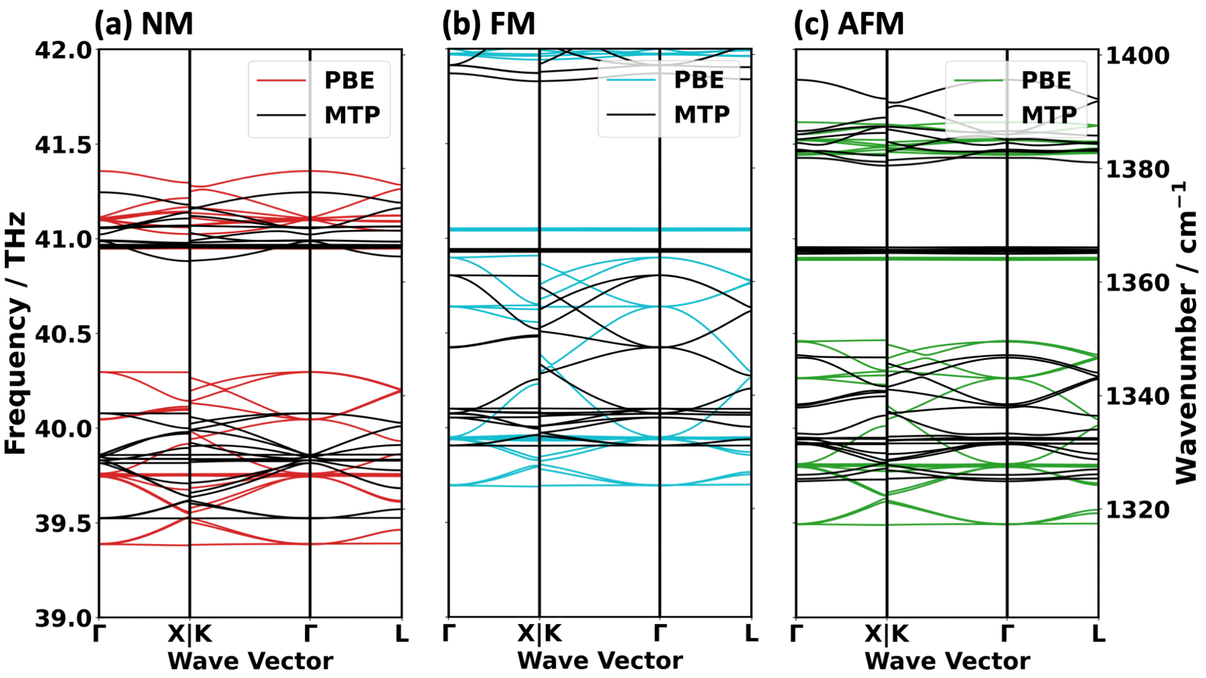

Figure S3. Phonon band structure comparison between the phonon modes in the frequency region from 39 THz to 42 THz of HKUST-1 in the NM (**a**), FM (**b**) and AFM states (**c**). The DFT/PBE results are shown with colored lines and the predictions made by the spin-dependent MTPs are added using black lines on top.

S4. Eigenvector overlaps of Γ-point phonons between DFT and machine-learned force fields for HKUST-1

For a comprehensive and visual comparison of the agreement between individual phonons at the Γ-point for the different spin configurations of HKUST-1, we plotted the Γ-point frequencies characterizing these phonon modes. They have been obtained using DFT/PBE and the spin-dependent MTPs are depicted as vertical lines in Figure S4-S9. In addition, the eigenvector overlaps of the frequencies were calculated based on their dot products [52] and a connecting line was drawn between the pair of phonon frequencies having the highest overlap. In the different figures, this was done for different frequency ranges. Overall, the results demonstrate a remarkable high level of agreement between the DFT/PBE and MTP results in terms of spin-dependent frequency shifts in all spectral regions.. This underscores the success of training and utilizing the MTPs for accurately predicting differences in phonon bands due to varying spin configurations.

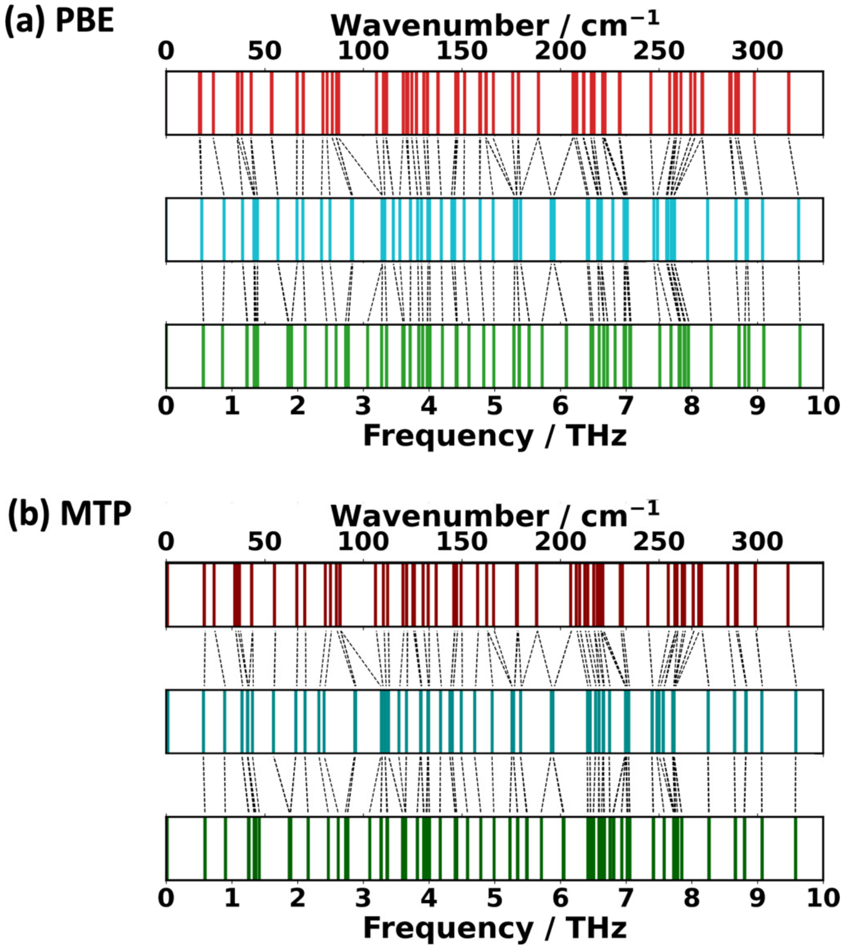

Figure S4. DFT/PBE- and MTP-calculated spin-dependent frequency shifts at Γ within the spectral region between 0 THz and 10 THz. The frequencies of the modes are plotted as vertical lines for DFT/PBE (**a**) and for the respective (**b**). Vibrations with the highest eigenvector overlaps are connected with grey dashed lines in these plots.

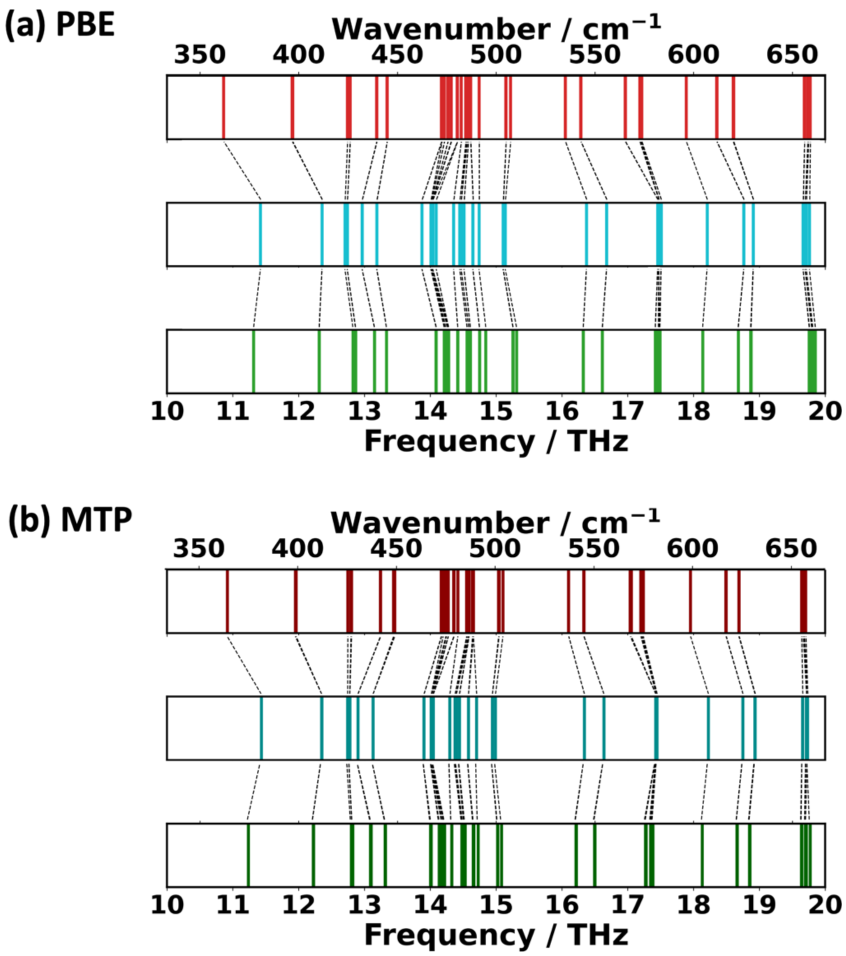

Figure S5. DFT/PBE- and MTP-calculated spin-dependent frequency shifts at Γ within the spectral region between 10 THz and 20 THz. The frequencies of the modes are plotted as vertical lines for DFT/PBE (**a**) and for the respective (**b**). Vibrations with the highest eigenvector overlaps are connected with grey dashed lines in these plots.

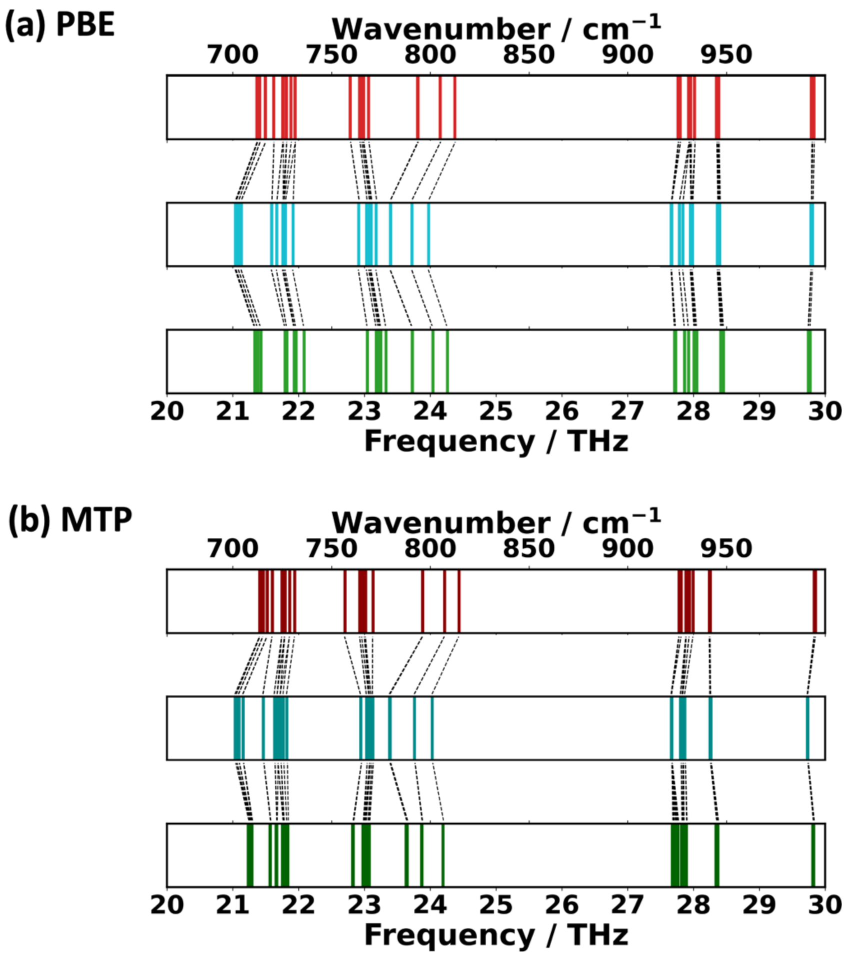

Figure S6. DFT/PBE- and MTP-calculated spin-dependent frequency shifts at Γ within the spectral region between 20 THz and 30 THz. The frequencies of the modes are plotted as vertical lines for DFT/PBE (**a**) and for the respective (**b**). Vibrations with the highest eigenvector overlaps are connected with grey dashed lines in these plots.

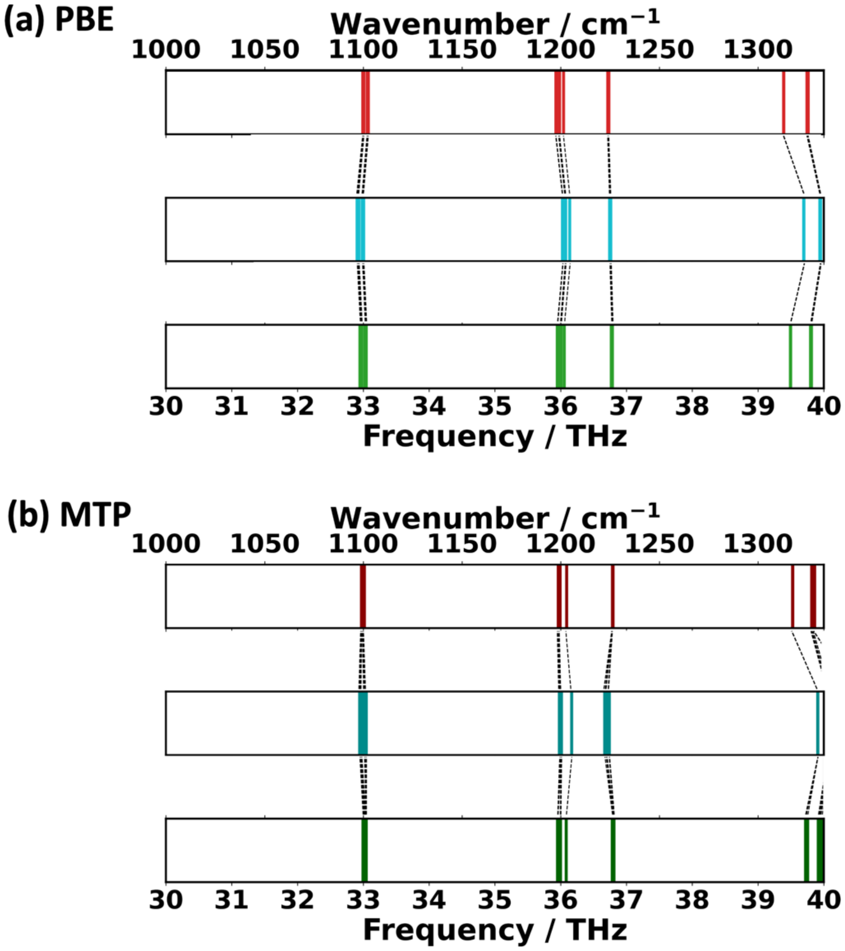

Figure S7. DFT/PBE- and MTP-calculated spin-dependent frequency shifts at Γ within the spectral region between 30 THz and 40 THz. The frequencies of the modes are plotted as vertical lines for DFT/PBE (**a**) and for the respective (**b**). Vibrations with the highest eigenvector overlaps are connected with grey dashed lines in these plots.

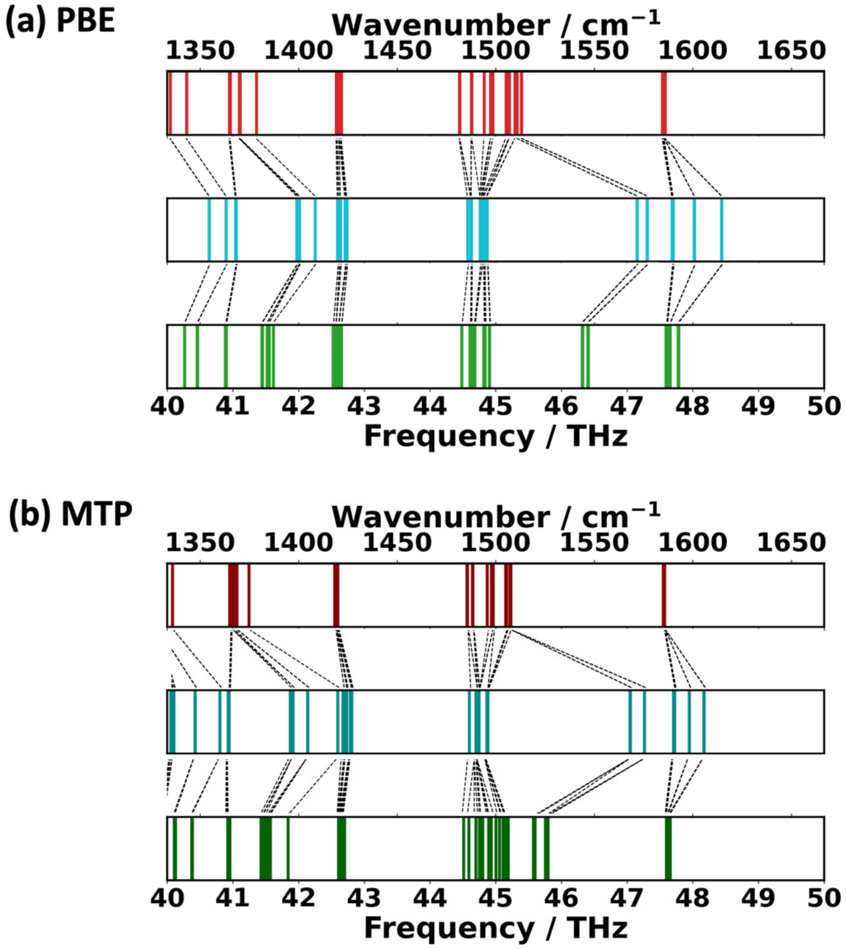

Figure S8. DFT/PBE- and MTP-calculated spin-dependent frequency shifts at Γ within the spectral region between 40 THz and 50 THz. The frequencies of the modes are plotted as vertical lines for DFT/PBE (**a**) and for the respective (**b**). Vibrations with the highest eigenvector overlaps are connected with grey dashed lines in these plots.

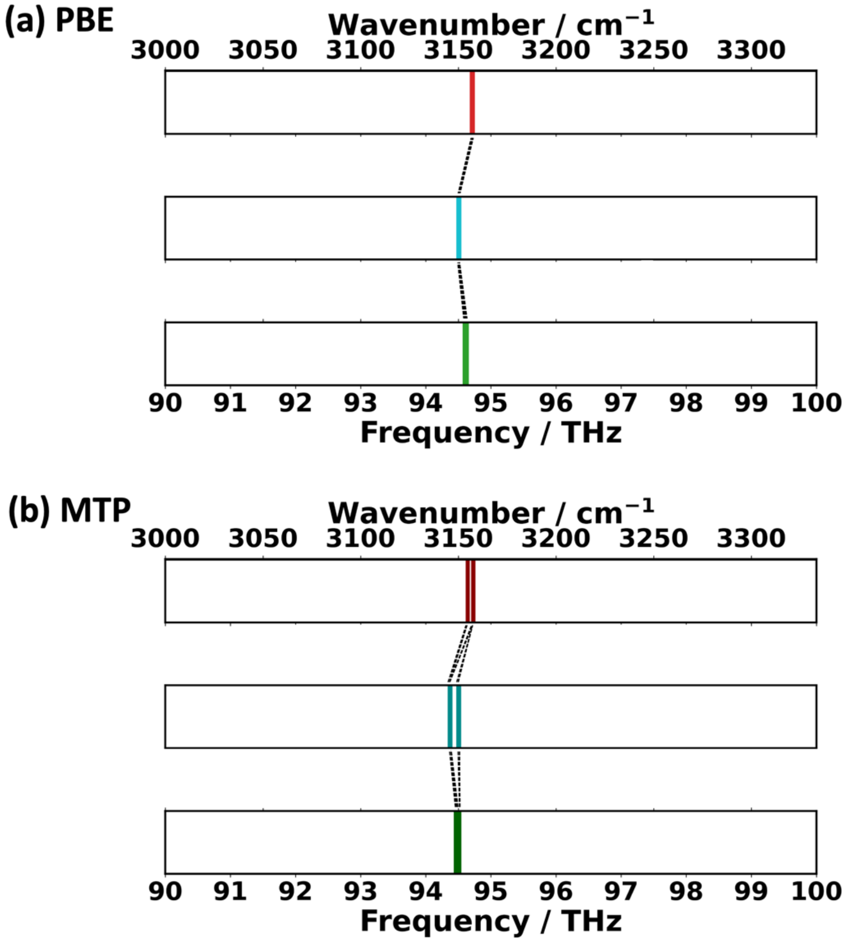

Figure S9. DFT/PBE- and MTP-calculated spin-dependent frequency shifts at Γ within the spectral region between 90 THz and 100 THz. The frequencies of the modes are plotted as vertical lines for DFT/PBE (**a**) and for the respective (**b**). Vibrations with the highest eigenvector overlaps are connected with grey dashed lines in these plots.

S5. Convergence tests on the total energy regarding the plane-wave cutoff and the k-grid

For the following convergence tests the total energy of the system per atom was used as criterion. The geometries for the three spin configurations of HKUST-1 were optimized with VASP [44] with computational settings (apart from the plane-wave cutoff and the k-point grid) as described in the Methods section of the main manuscript.. Panel (a) of Figure S10 shows the dependence of the total energy per atom for HKUST-1 in the FM and AFM magnetic states for a 1×1×1 Γ-centered k-point grid for the electronic states as a function of the energy cutoff of the plane-wave basis set. In panel (b) the cutoff is fixed at 900 eV and the dependence on the (Γ-centered) k-rid is shown. Convergence is considered to be achieved, once the total energy change from compared to the previous setting is less than 0.05 meV/atom, while at the same time, the difference compared to the highest setting is clearly below 0.1 meV/atom. Correspondingly, 900 eV energy cutoff and a 1×1×1 Γ-centered k-point grid are considered to be converged.

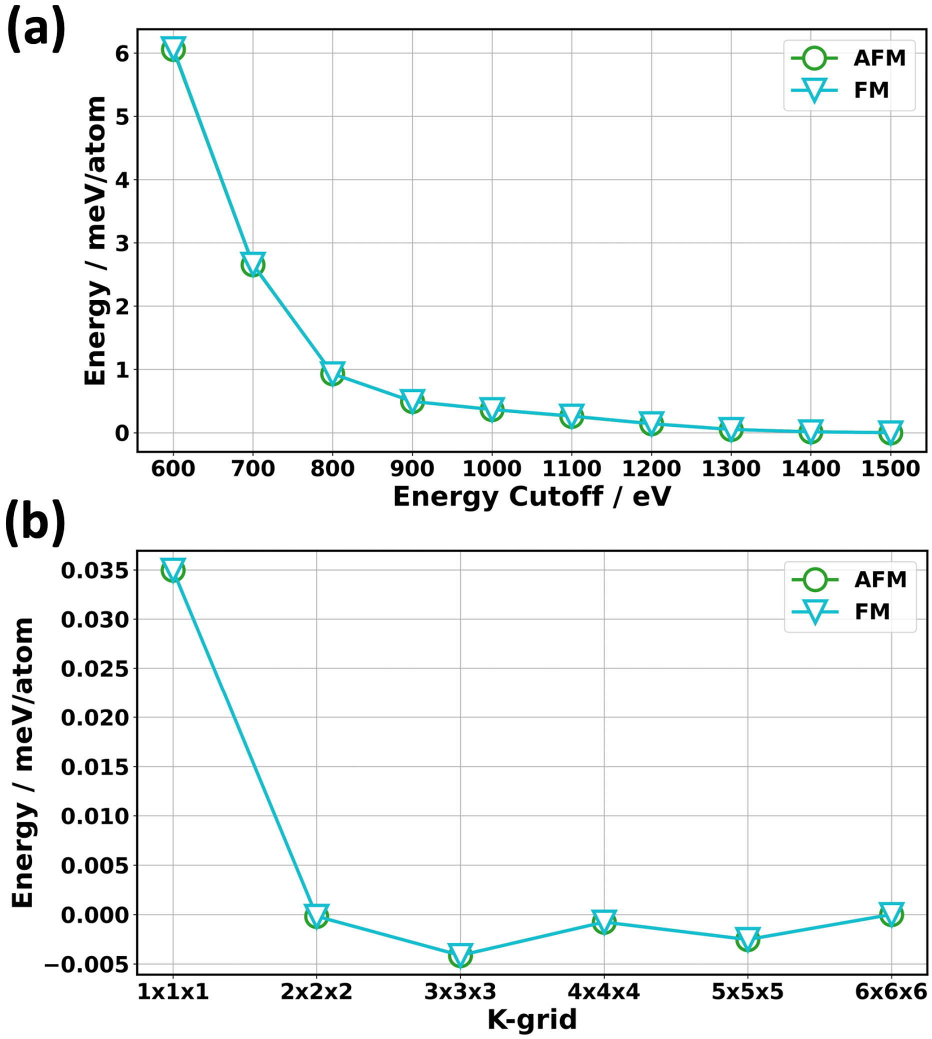

Figure S10. Convergence tests for the AFM and FM states of HKUST-1 based on (changes) in the total energy per atom. In panel (**a**) that quantity is plotted as a function of the energy cutoff of the plane-waves used in VASP for a 1×1×1 Γ-centered k-point grid. In panel (**b**) the energy cutoff is fixed at 900 eV and the k-grid is varied. The marginal variations of the energy per atom in that panel are a consequence of the entirely flat electronic bands of HKUST-1. The values of the total energy per atom are given relate to the final data point.

S6. Convergence of the supercell size of HKUST-1 for phonon calculations using machine-learned force fields

Due to the impossibility of performing supercell convergence tests for phonons of HKUST-1 using DFT/PBE with converged settings, (given that the primitive trigonal unit cell already contains 156 atoms and a 2×2×2 supercell would, for example, contain 1248 atoms) we performed these tests using the spin-dependent and system-specifically trained MTPs, even though these are hampered by the relatively short radial cutoff. In Figure S11 and Figure S12 we compare the Γ-point frequencies for 1×1×1, 2×2×2, and 3×3×3 supercells. The phonon frequencies are identical, which we attribute to the large primitive unit cell of HKUST-1 such that in

conjunction with the radial cutoff of the MTPs supercells do not provide any new information. This is also observed for the phonon bands in the low-frequency region in Figure S13.

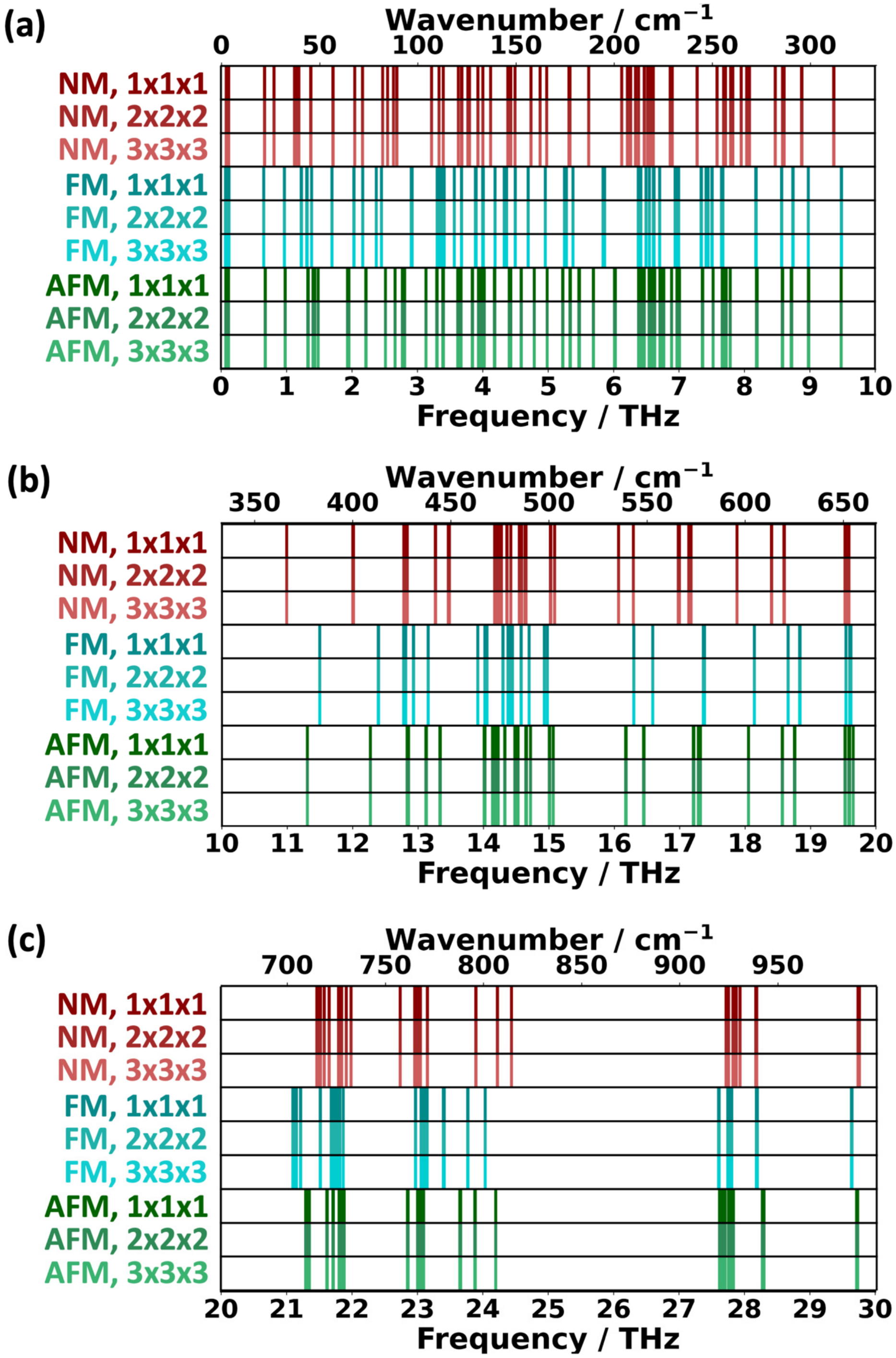

Figure S11. Phonon modes at the Γ-point in different frequency intervals ranging from 0 THz to 10 THz (**a**), from 10 THz to 20 THz (**b**), and from 20 THz to 30 THz (**c**), for the NM (red), FM (blue) and AFM (green) states of HKUST-1 plotted as vertical lines and calculated for different supercell sizes using MTPs.

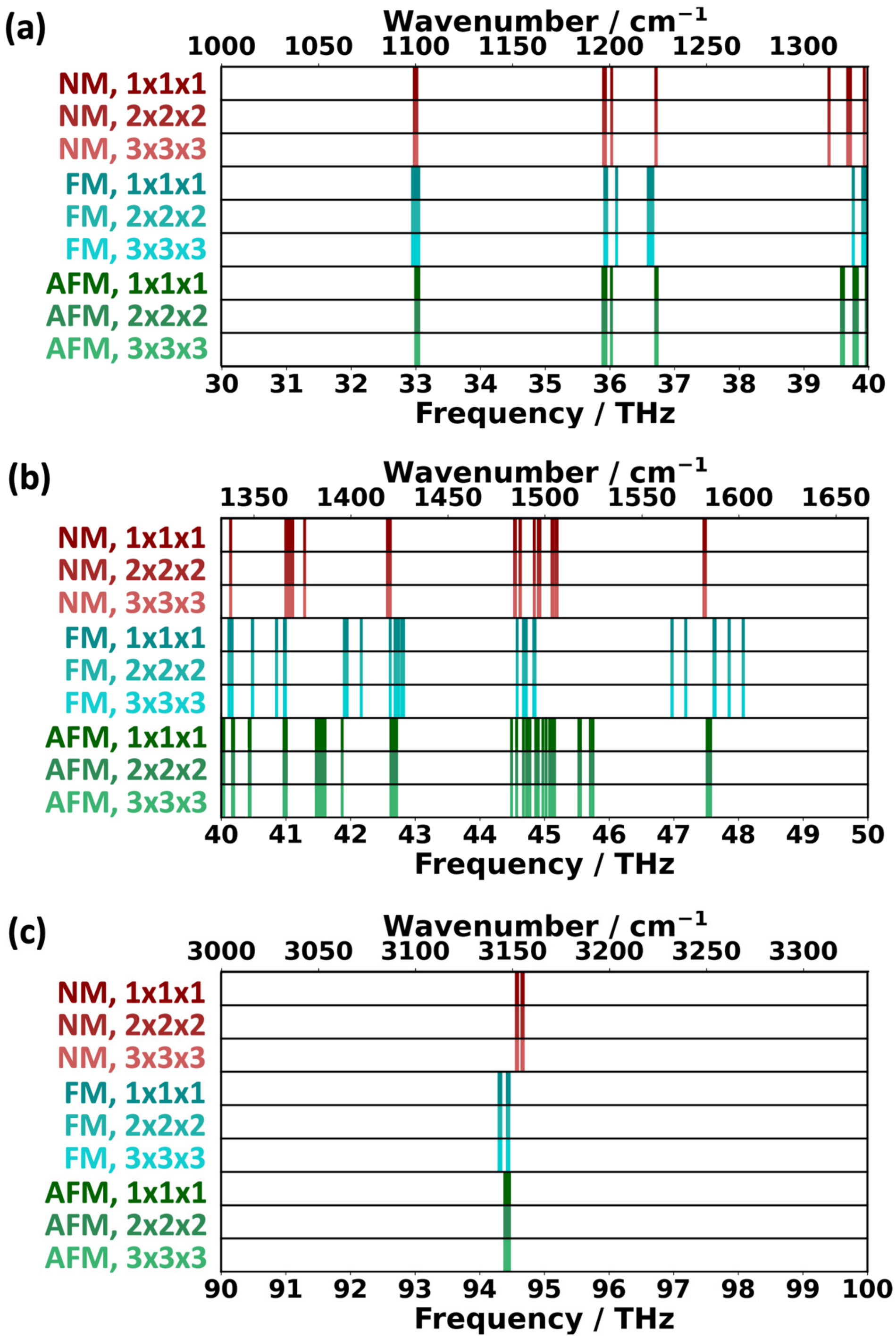

Figure S12. Phonon modes at the Γ-point in different frequency intervals ranging from 30 THz to 40 THz (**a**), from 40 THz to 50 THz (**b**), and from 90 THz to 100 THz (**c**), for the NM (red), FM (blue) and AFM (green) states of HKUST-1 plotted as vertical lines and calculated for different supercell sizes using MTPs.

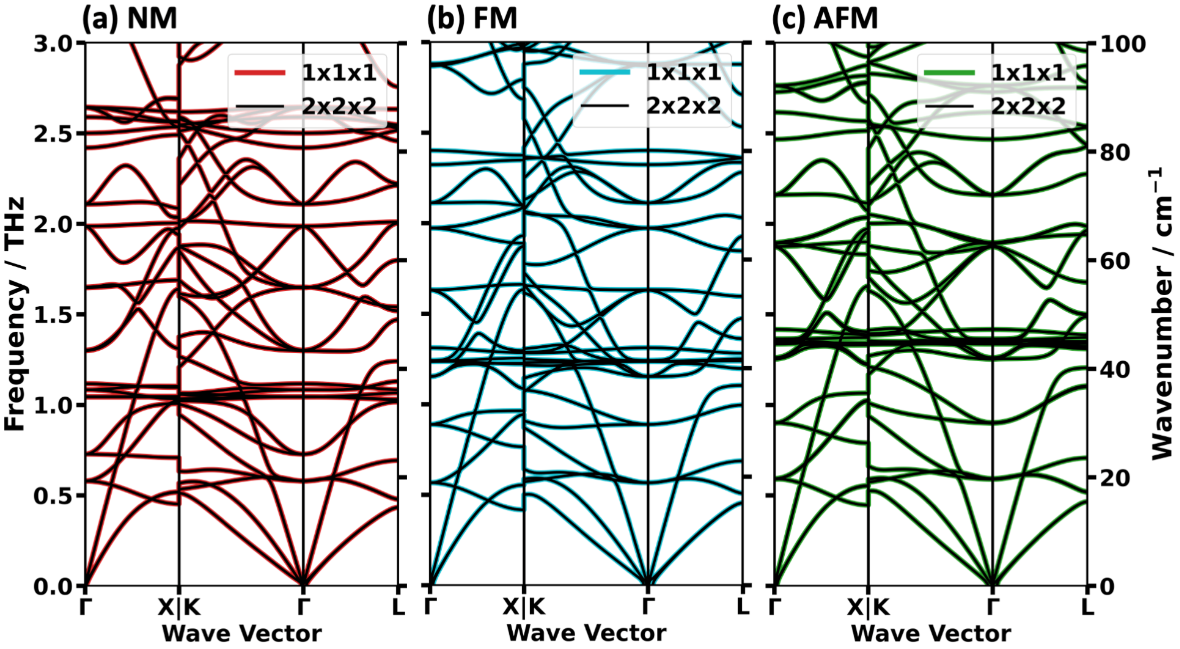

Figure S13. Phonon band structure comparison of HKUST-1 in the NM (**a**), FM (**b**) and AFM (**c**) states calculated with MTPs using the primitive unit cell (1×1×1) and the 2×2×2 supercell.

S7. Performance of the MTPs

Out of the five trained MTPs for each spin configuration, for the data shown in the main manuscript the one with the best cost function has been picked. In order to show that independent of this selection, each of the parametrized MTPs would provide a highly satisfactory description of the spin-dependent phonon properties of HKUST-1, the following tables contain the root mean square deviations of the Γ-point frequencies (wavenumbers) of equivalent modes calculated with each of the MTPs and with DFT/PBE. In Table S2, the RMSDs for the most spin-sensitive frequency region between 38 THz and 50 THz is reported, while in Table S3 the values for the entire range of frequencies present in HKUST-1 are listed. The MTPs that produced lowest value for the cost function are printed in bold. For the NM state this MTP is also the one that produced the lowest RMSD value. However, for the spin-polarized solutions (FM and AFM) this not the case and picking other MTPs would have provided an even better agreement to the DFT data. Still, the calculated RMSD values for different MTPs are rather similar such that a more sophisticated pick would not have caused dramatic changes.

In passing we note that the specific choice of the MTP can also determine, which mode displays particularly large deviations from the DFT reference. For example, as discussed in the main manuscript, when picking the 'ideal MTP' based on the smallest loss function, the deviations from the DFT-calculated modes around 46.3 THz in the AFM state are rather large and the MTP puts them at 45.9 THz. When picking some of the other MTPs for that spin configuration that deviation would decrease, while other deviations would increase. Thus, larger deviations for specific modes are not necessarily only related to the nature of those modes but are also impacted by the stochastic initialization of the MTP parametrization and the choice of the 'best MTP'.

Table S3. RMSDs between frequencies (wavenumbers) of equivalent Γ-point vibrations of HKUST-1 calculated using DFT/PBE (enforcing certain spin configurations) and calculated with spin-sensitively parametrized MTPs for the frequency range between 38 THz and 50 THz. The values for the MTPs producing the lowest cost function for each spin state are highlighted in bold. They have been used for calculating the data contained in the main manuscript.

MTP	NM	FM	AFM
**1**	0.086 THz (2.86 cm^{-1})	0.168 THz (5.61 cm^{-1})	0.235 THz (7.83 cm^{-1})
**2**	0.107 THz (3.56 cm^{-1})	**0.165 THz (5.46 cm^{-1})**	0.226 THz (7.53 cm^{-1})
**3**	0.097 THz (3.24 cm^{-1})	0.121 THz (4.03 cm^{-1})	**0.210 THz (7.00 cm^{-1})**
**4**	0.091 THz (3.04 cm^{-1})	0.106 THz (3.55 cm^{-1})	0.223 THz (7.44 cm^{-1})
**5**	**0.075 THz (2.47 cm^{-1})**	0.113 THz (3.77 cm^{-1})	0.239 THz (7.97 cm^{-1})

Table S4. RMSDs between frequencies (wavenumbers) of equivalent Γ-point vibrations of HKUST-1 calculated using DFT/PBE (enforcing certain spin configurations) and calculated with spin-sensitively parametrized MTPs for the frequency range between 0 THz and 100 THz. The values for the MTPs producing the lowest cost function for each spin state are highlighted in bold. They have been used for calculating the data contained in the main manuscript.

MTP	NM	FM	AFM
**1**	0.058 THz (1.94 cm^{-1})	0.123 THz (4.10 cm^{-1})	0.130 THz (4.33 cm^{-1})
**2**	0.063 THz (2.10 cm^{-1})	**0.105 THz (3.50 cm^{-1})**	0.121 THz (4.04 cm^{-1})
**3**	0.057 THz (1.88 cm^{-1})	0.072 THz (2.41 cm^{-1})	**0.127 THz (4.24 cm^{-1})**
**4**	0.055 THz (1.83 cm^{-1})	0.069 THz (2.30 cm^{-1})	0.121 THz (4.04 cm^{-1})
**5**	**0.052 THz (1.74 cm^{-1})**	0.062 THz (2.06 cm^{-1})	0.132 THz (4.42 cm^{-1})

S8. Comparison of PBE-derived phonon frequencies calculated with VASP and FHI-aims including the impact of the van der Waals correction

To expand the range of tested van der Waals corrections and to later efficiently perform hybrid-functional calculations, in addition to the VASP simulations discussed in the main manuscript, also simulations using the FHI-aims code [62] were performed. For the PBE-based simulations the default intermediate and tight basis atom-centered basis sets of FHI-aims were used. A 1×1×1 k-grid centered at Γ was employed together with a 2×2×2 grid for the many-body dispersion (MBD) correction [66], which was used as an a posteriori van

der Waals correction in the FHI-aims calculations. As FHI-aims is an all-electron code, scalar relativistic effects were accounted for using the ZORA formalism [86]. Geometries were optimized using the Broyden-Fletcher-Shanno-Goldfarb algorithm enhanced by the trust radius method [62], with a tolerance threshold of 10^{-2} eV·Å^{-1}. The frequencies of the Γ-point phonons calculated with FHI-aims with tight and intermediate basis sets using MBD and non van der Waals corrections and with VASP employing different energy cutoffs for the PW basis setts and using no, the D3 [56], and the D4 [63–65] van der Waals corrections are compared in the following figures. In passing we note that the unit cell of HKUST-1 was relaxed using the same approach that was employed for the subsequent phonon calculations.

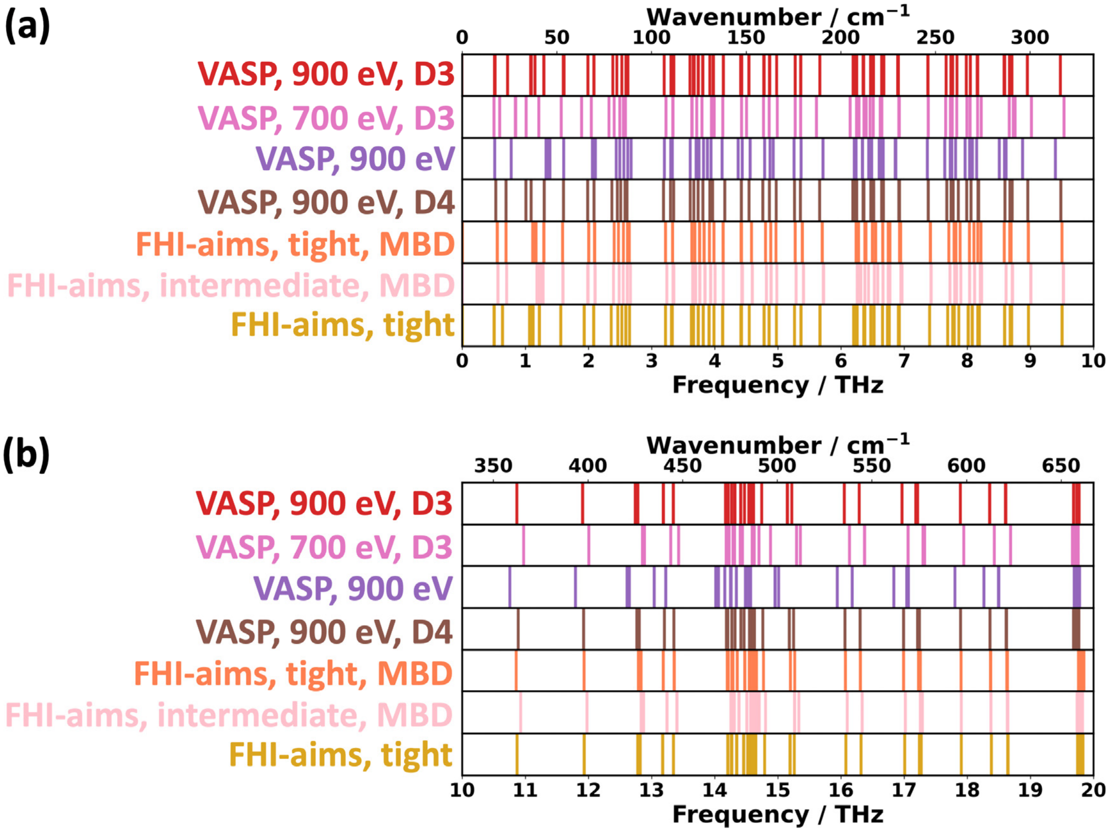

Figure S14. DFT calculated frequencies of Γ-point phonons of HKUST-1 in the NM state between 0 THz and 10 THz **(a)** as well as between 10 and 20 THz **(b)** calculated with the VASP code: using a 900 eV cutoff and the D3 van der Waals correction (line 1) a 700 eV cutoff and the D3 van der Waals correction (line 2), a 900 eV cutoff and no van der Waals correction (line 3), and a 900 eV cutoff and the D4 van der Waals correction (line 4). FHI-aims calculations have been performed employing a tight basis set and the MBD van der Waals correction (line 5), an intermediate basis set and the MBD van der Waals correction (line 6), and a tight basis set and no van der Waals correction (line 7).

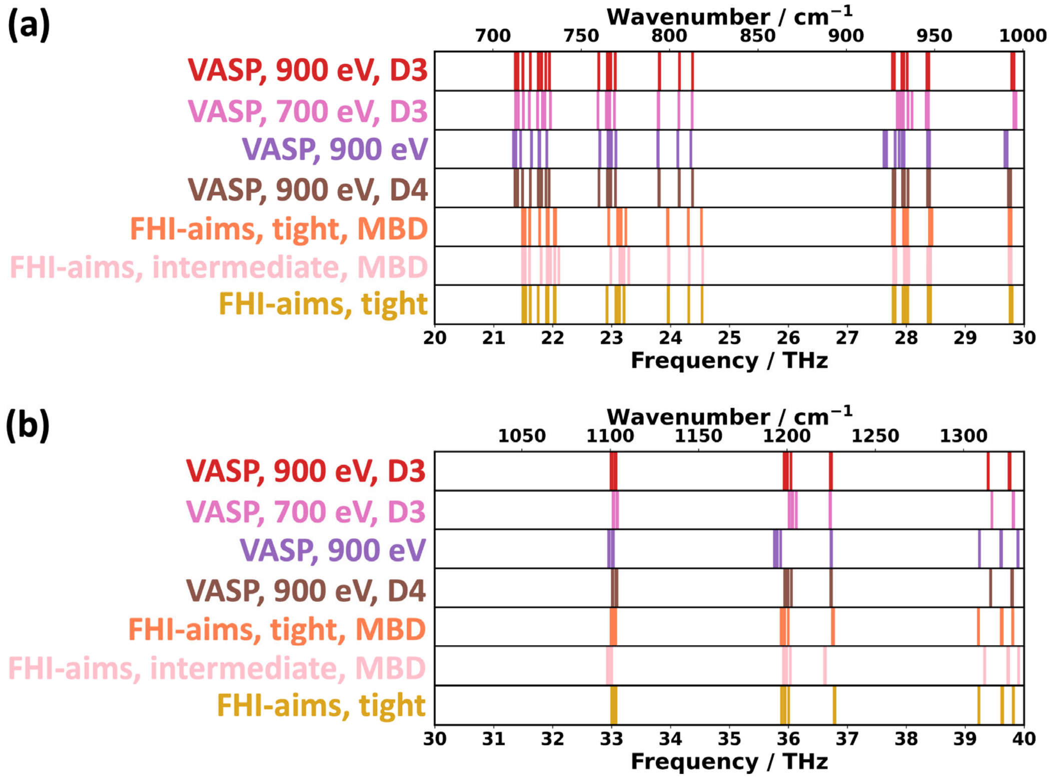

Figure S15. DFT calculated frequencies of Γ-point phonons of HKUST-1 in the NM state between 20 THz and 30 THz (**a**) as well as between 30 and 40 THz (**b**) calculated with the VASP code: using a 900 eV cutoff and the D3 van der Waals correction (line 1) a 700 eV cutoff and the D3 van der Waals correction (line 2), a 900 eV cutoff and no van der Waals correction (line 3), and a 900 eV cutoff and the D4 van der Waals correction (line 4). FHI-aims calculations have been performed employing a tight basis set and the MBD van der Waals correction (line 5), an intermediate basis set and the MBD van der Waals correction (line 6), and a tight basis set and no van der Waals correction (line 7).

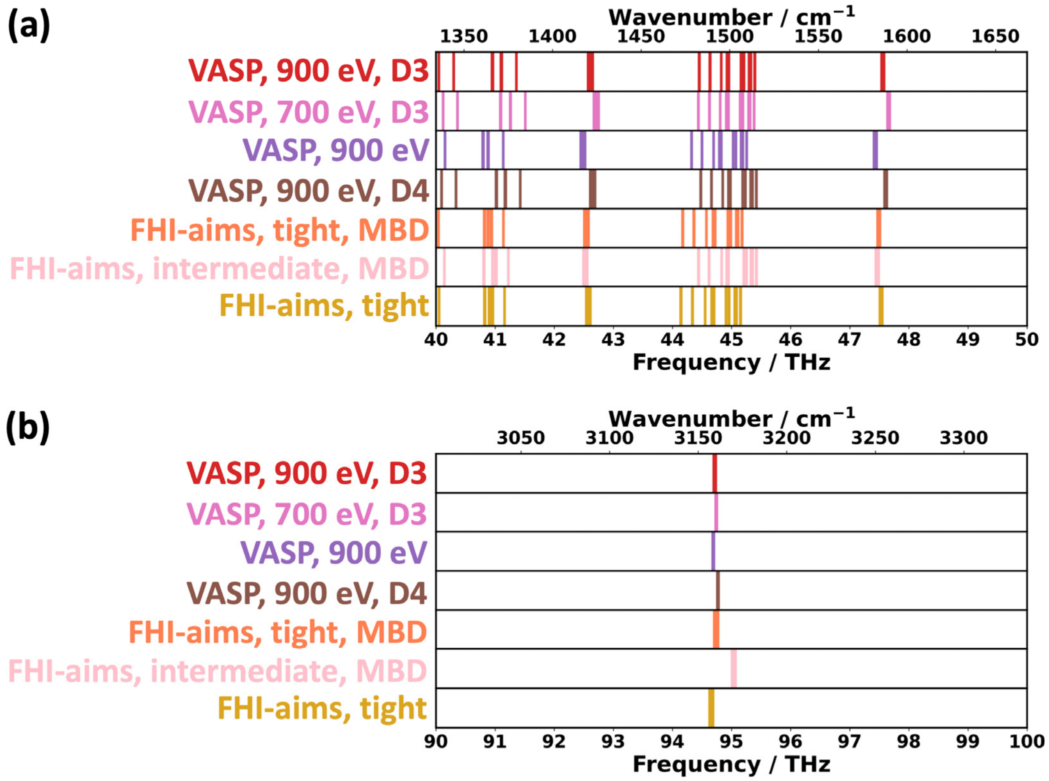

Figure S16. DFT calculated frequencies of Γ-point phonons of HKUST-1 in the NM state between 40 THz and 50 THz (**a**) as well as between 90 and 100 THz (**b**) calculated with the VASP code: using a 900 eV cutoff and the D3 van der Waals correction (line 1) a 700 eV cutoff and the D3 van der Waals correction (line 2), a 900 eV cutoff and no van der Waals correction (line 3), and a 900 eV cutoff and the D4 van der Waals correction (line 4). FHI-aims calculations have been performed employing a tight basis set and the MBD van der Waals correction (line 5), an intermediate basis set and the MBD van der Waals correction (line 6), and a tight basis set and no van der Waals correction (line 7).

This comparison shows that the overall trends are for the phonon frequencies are the same for all calculations. Nevertheless, one also sees certain differences: for both codes picking a too small basis set (700 eV cutoff and intermediate) results in most instances in somewhat too high phonon frequencies. Comparing the VASP calculations with D3 and D4 van der Waals corrections, the phonon frequencies are essentially identical. Entirely disregarding van der Waals interactions results in some larger deviations compared to the VASP/D3 calculations, while it has only a rather minor impact compared to the FHI-aims/MBD case. Concerning the comparison between the VASP/D3 or VASP/D4 and the FHI-aims/MBD calculations, the general trends are recovered, but especially between 20 and 25 THz and between 40 and 50 THz some shifts are observed. Overall, these data show that both the choice of the van der Waals correction as well as convergence of the basis set do have a non-negligible impact on the phonon frequencies, although they do not cause qualitative deviations.

Notably, despite these variations between the VASP/D3 and the FHI-aims/MBD simulations, they are still to minor to have a profound impact on the spin-dependence of the DOSs and the infrared spectra, as shown in Figures S17 and S18.

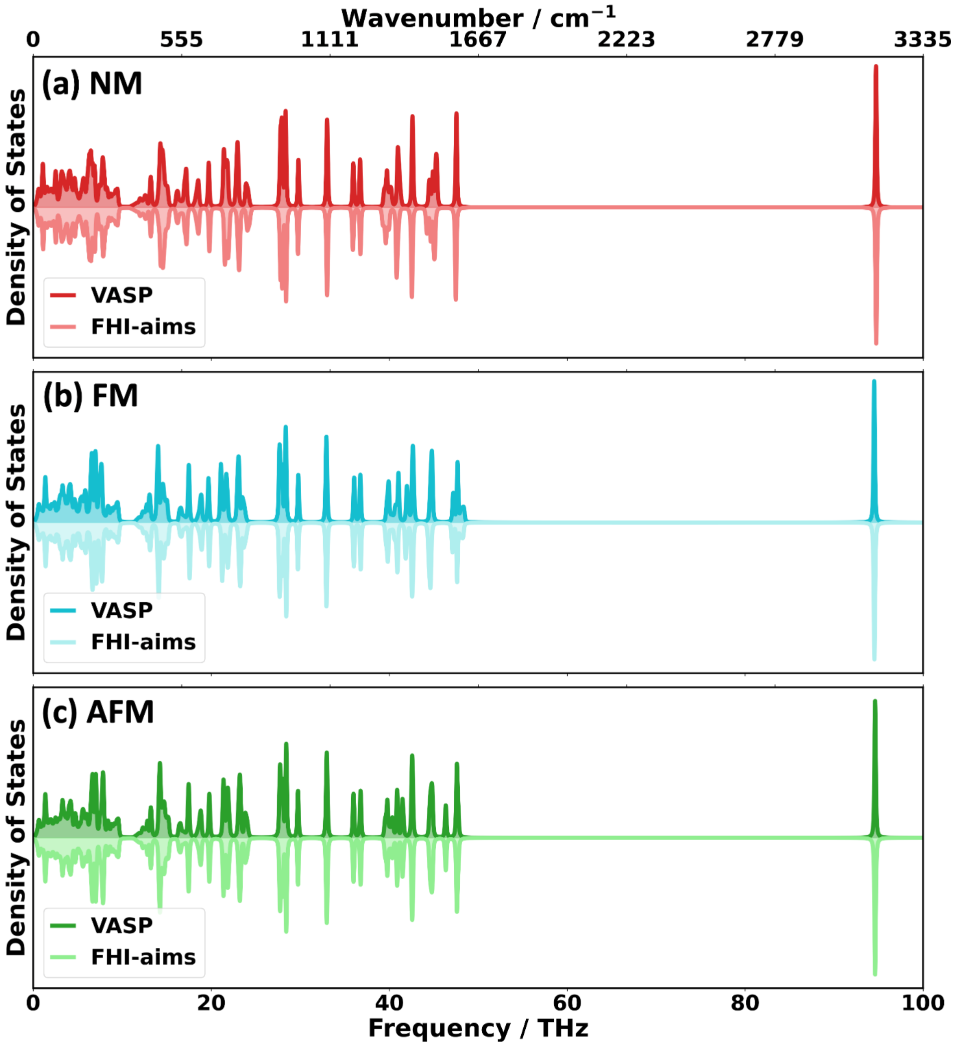

Figure S17. Spin-state dependence of the VASP/D3 and FHI-aims/MBD calculated DOSs for the PBE functional obtained using a 10×10×10 q-mesh for the NM (**a**), FM (**b**) and AFM (**c**) states of HKUST-1. The top DOSs shows the results obtained with VASP, while the bottom curves display the ones calculated with FHI-aims.

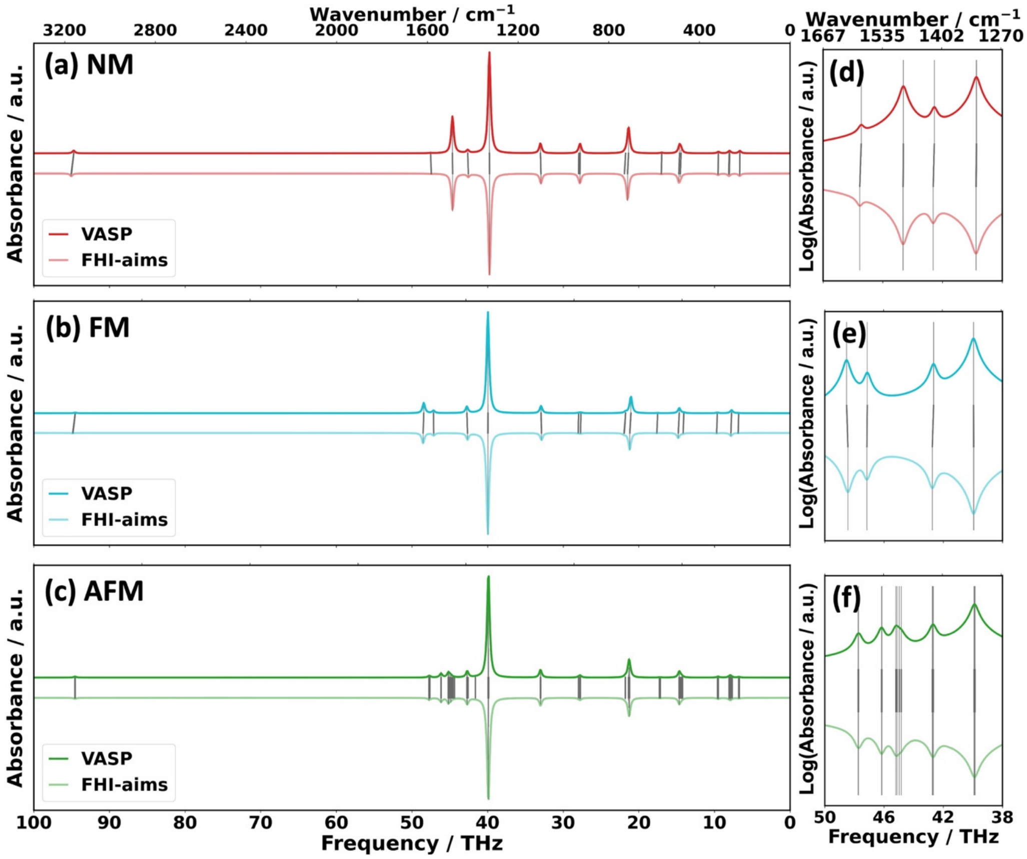

Figure S18. Spin-state dependence of the VASP/D3 and FHI-aims/MBD calculated IR spectra using the PBE functional for the NM (**a**), FM (**b**) and AFM (**c**) states of HKUST-1. The top spectra show the results obtained with VASP, while the bottom curves display the ones calculated with FHI-aims. The calculation of the IR spectra is described in the last section of the ESI.

S9. Impact of using a hybrid functional on phonon frequencies

Considering the increased computational demands when employing the PBE0 [57] functional, in the corresponding FHI-aims calculations the light basis set had to be used. This basis set cannot be considered as being converged, but the use of larger basis sets is prevented by the complexity and size of the unit cell of HKUST-1. Therefore, the following data have to be taken with a grain of salt and can only serve for a qualitative comparison. The overview plot in Figure S19 indicates that the overall-shapes of the spin-dependent DOSs largely prevail in the PBE0 calculations with the main effect being a shift of the DOS features to higher frequencies. That shift is clearly more pronounced at higher frequencies. Not unexpectedly, an equivalent shift increasing with frequency is also observed, when comparing the IR-spectra in Figure S20. On peculiarity of the data shown in this plot is that the functional-dependent shifts in the IR-active modes around 16 THz and around 22 THz appear more pronounced for the AFM state.

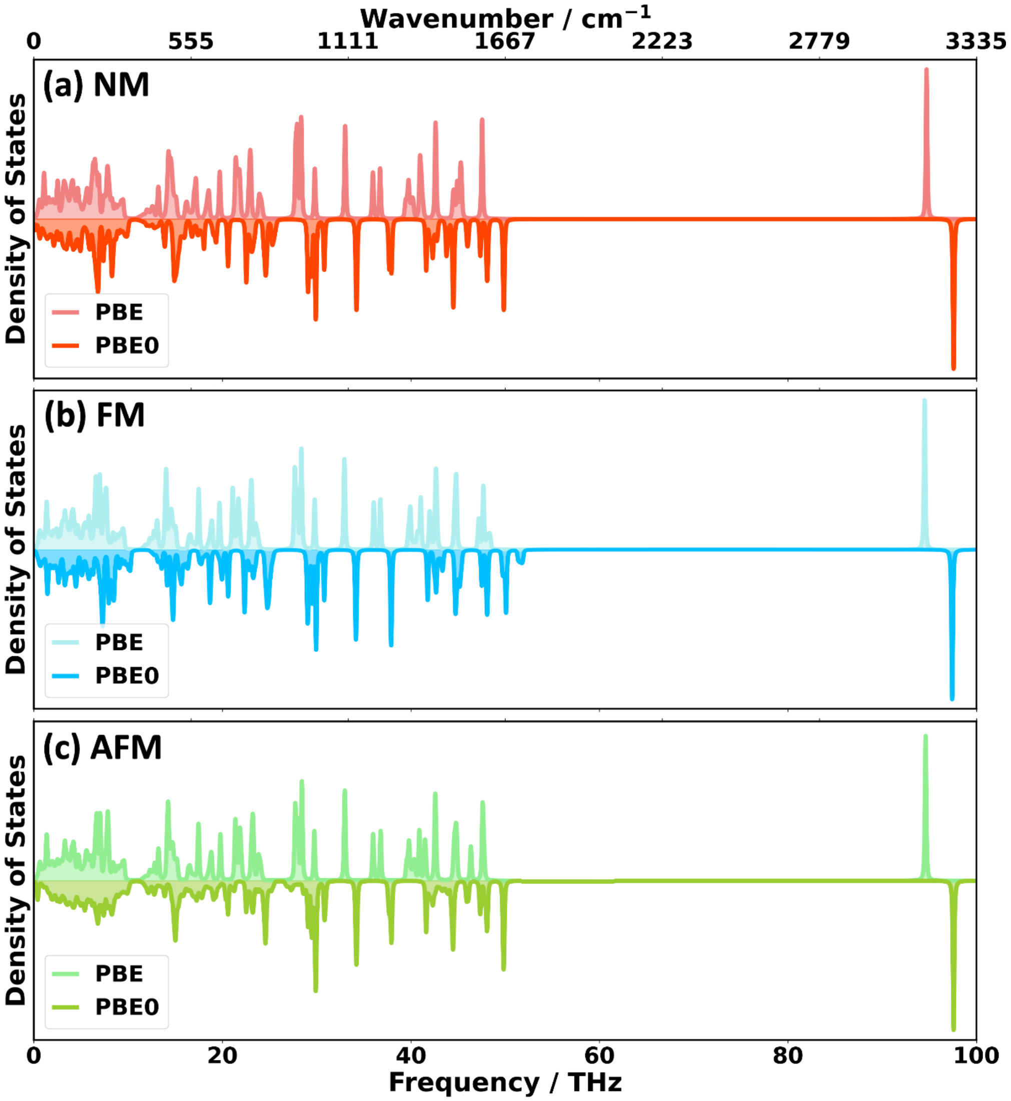

Figure S19. Spin-state dependence of the FHI-aims/MBD calculated DOSs employing the PBE and the PBE0 functionals (obtained using a 10×10×10 q-mesh) for the NM (**a**), FM (**b**) and AFM (**c**) states of HKUST-1. The

top DOSs show the results obtained with the PBE functional, while the bottom curved display the ones obtained using PBE0.

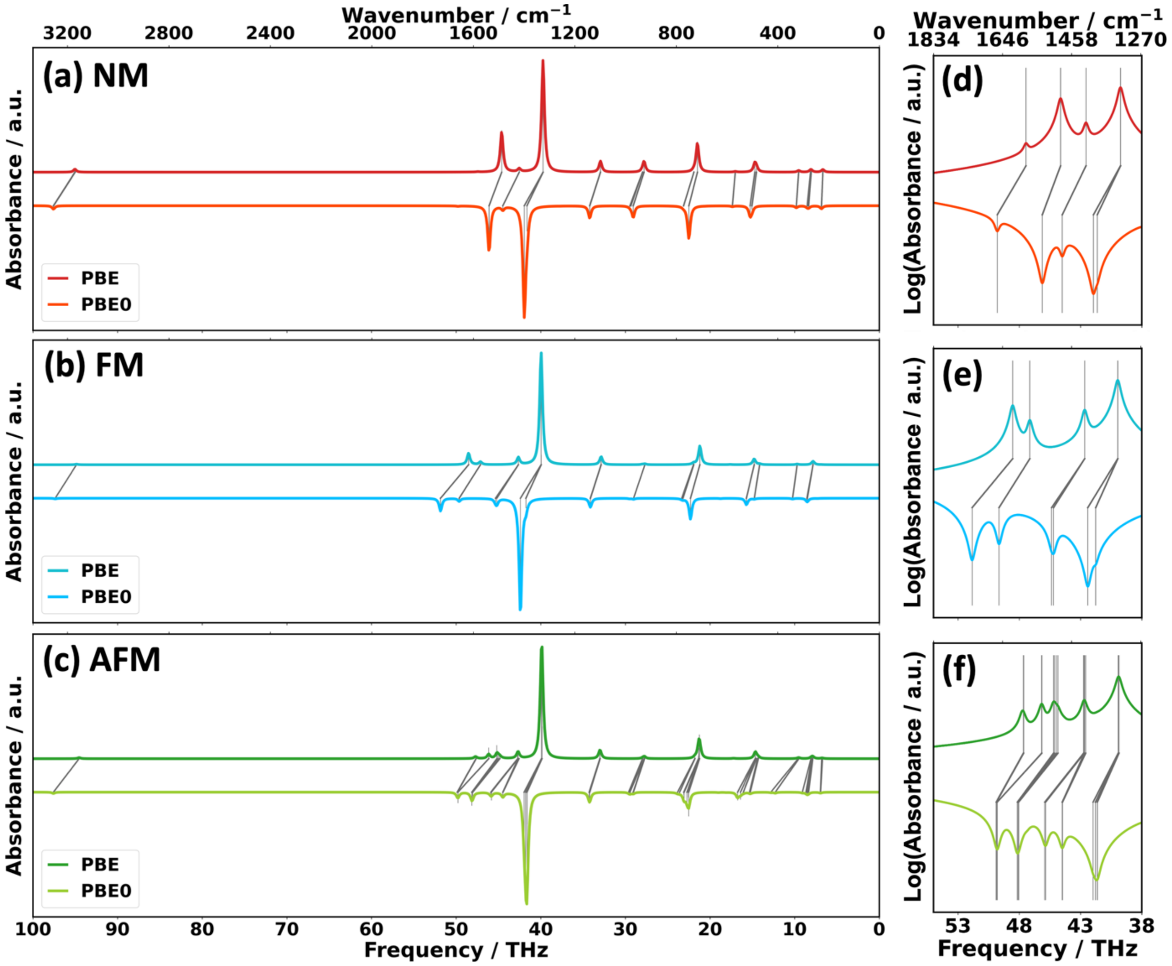

Figure S20. Spin-state dependence of the FHI-aims/MBD calculated IR spectra for the NM (**a**), FM (**b**) and AFM (**c**) states of HKUST-1 calculated with the PBE and PBE0 functionals. The top spectra show the results obtained with VASP, while the bottom curves display the ones calculated with FHI-aims. The right panels show a zoom into the region between 38 THz and 55 THz. The calculation of the IR spectra is described in the last section of the ESI.

Notably, also the spin-dependent differences in the shapes of the IR spectra in the most spin-affected region between 38 THz and 55 THz prevail, even though especially for the AFM state this is somewhat coincidentally caused by several peaks displaying larger and others displaying smaller shifts. This is also visible when considering all states in that region (rather than only the IR-active ones), as is done in Figure S21. Overall, this graph, however, confirms the notion that the main impact of using a hybrid functional is a shift

to higher frequencies, where due to the very small basis set in the PBE0 calculations a detailed comparison of the relative shifts of individual Γ-point phonons appears futile.

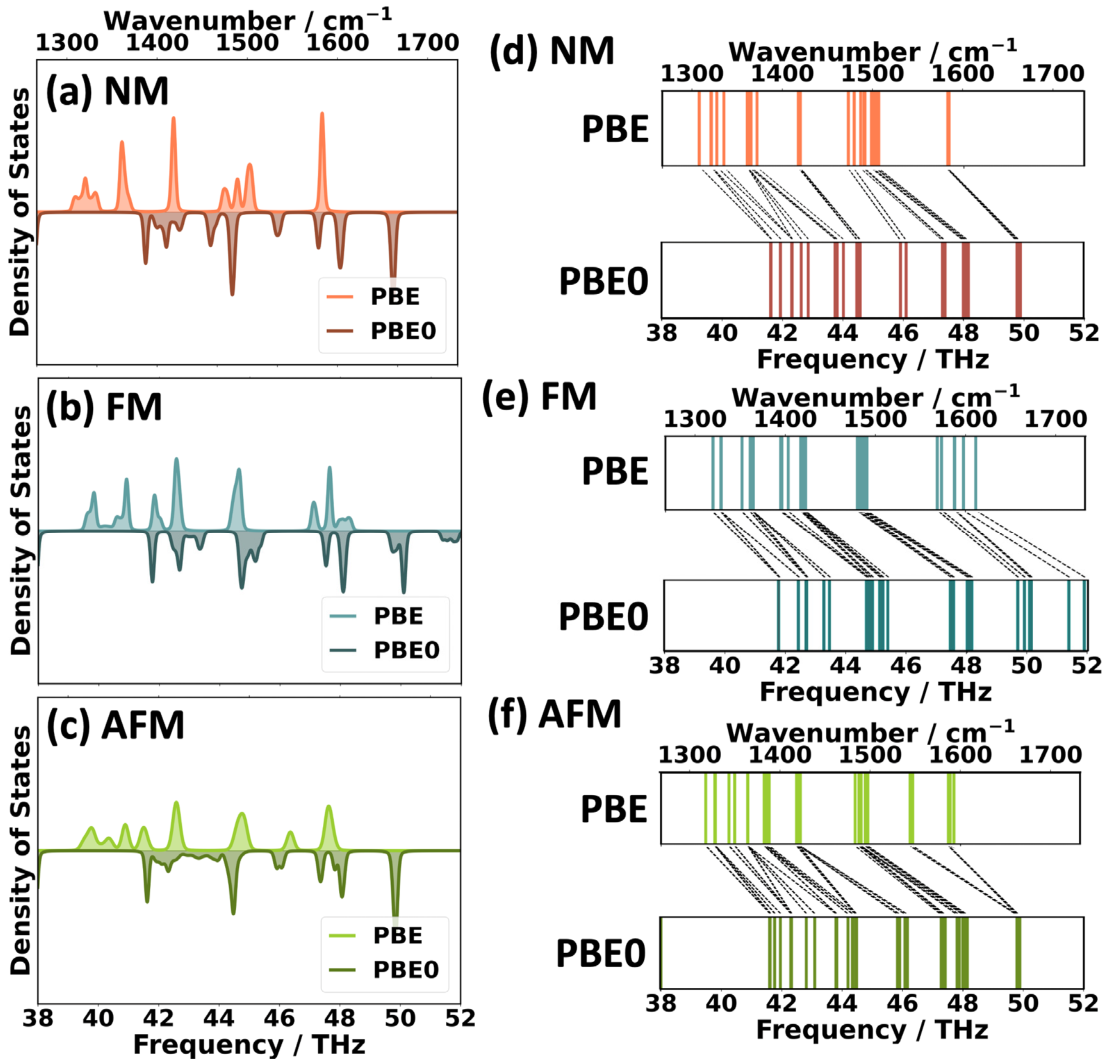

Figure S21. Phonon DOSs for the NM (red) (**a**), FM (blue) (**b**) and AFM (green) (**c**) configurations of HKUST-1 between 38 THz and 55 THz calculated with FHI-aims. The results obtained with DFT/PBE are plotted in the upper half of the graphs, while the results for the computations performed with DFT/PBE0 are displayed in the bottom half in a darker shade of the used color. Panels (**d**), (**e**) and (**f**) illustrate the shifts in frequencies of the Γ-point vibrations between the GGA and hybrid functional for each spin state. In these plots, vibrations with the highest eigenvector overlaps are connected by grey dashed lines.

S10. Comparison between simulated and experimental IR data

To calculate IR intensities, the Born effective charge tensor, which is accessible in VASP (version: 6.3.0) [44], was used. The Born effective charges are computed by applying a small, static electric field to the atoms and calculating the resulting induced polarization using density functional perturbation theory (DFPT). [87] This method is computationally efficient and accurate. In contrast, FHI-aims (version: 221103) [62] uses first order perturbation theory to calculate the Born effective charges. [88] This method is based on finite differences in the electronic density and is very expensive in terms of computational time, especially for large systems like HKUST-1. The reason for this is that the Born effective charges are calculated by solving the linear response of the electronic density to the perturbation, which requires diagonalization of a large matrix. Therefore, the computational cost scales with the cube of the number of atoms in the system, making it impractical for larger systems. Thus, to obtain an IR spectrum for FHI-aims, the corresponding frequencies from FHI-aims were combined with the IR intensities computed with VASP. This was done by calculating the eigenvector overlap between the VASP and FHI-aims computed vibrations and assigning identical intensities to the modes with the highest overlap. We found that there were no differences between the computed spin-dependent IR spectra using the PBE functional for VASP and FHI-aims (as shown in Figure S6) with respect to the frequencies of the vibrations. In passing it is noted that, as with VASP we only succeeded in converging calculations using the PBE functional, this means that also for the PBE0 calculations in FHI-aims, PBE intensities had to be used. We, however, do not expect that to pose a major problem.

Figures S23 and S24 show a comparison between the simulated IR spectra and experiments for the different spin configurations and calculated with the PBE and PBE0 functionals. Overall, the PBE data appear to underestimate the frequencies associated with the different peak positions resulting in a better fit of the PBE0 calculations. Also, the more pronounced shift of peak positions for the AFM state in the PBE0 calculations at frequencies around 16 THz (mentioned already in the context of Figures S20) is clearly visible. Still, it should be noted that these calculations suffer from the very small used basis set. Moreover, one clearly sees that the experimental spectra are not of sufficient quality to be able to distinguish between different spin states. In fact, even the coexistence of different spin states cannot be ruled out from the comparison between simulations and experiments. Regarding a comparison of peak intensities, it should be noted that in the simulations an isotropic sample has been assumed, which is not necessarily the case for the experiments. Moreover, in the experiments more peaks are visible than in the simulations. This might be due to the existence of axial ligands, solvent molecules, or unreacted monomers.

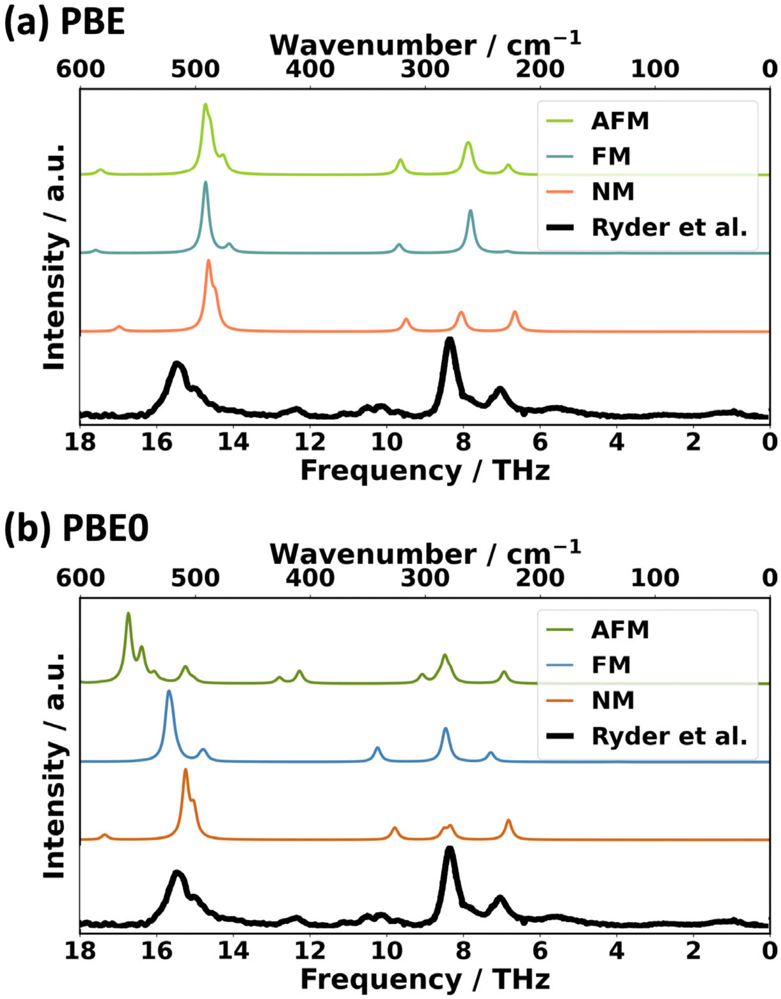

Figure S22. Comparison between simulated IR spectra of HKUST-1 in the AFM, FM and NM state for FHI-aims/MBD using the PBE functional (**a**) and the hybrid functional PBE0 (**b**) as well as experimental IR data [89] for the frequency range from 0 to 18 THz.

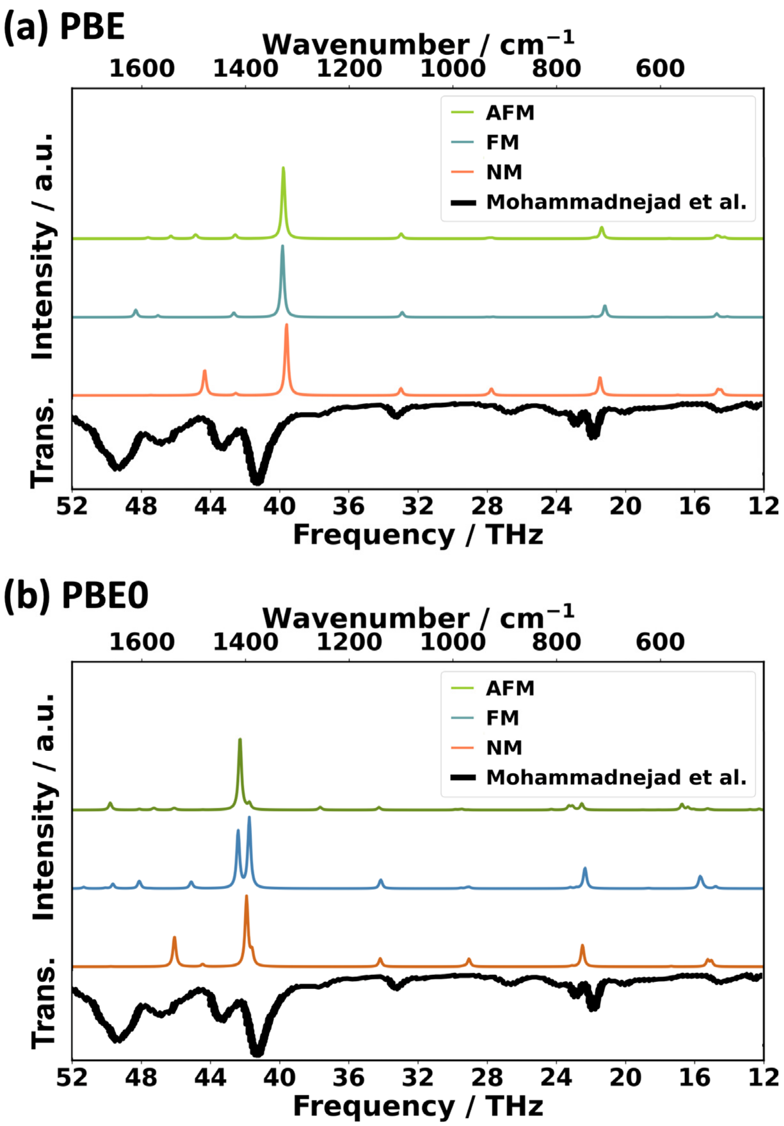

Figure S23. Comparison between simulated IR spectra of HKUST-1 in the AFM, FM and NM state for FHI-aims/MBD using the PBE functional (**a**) and the hybrid functional PBE0 (**b**) as well as experimental IR data [90] for the frequency range from 12 to 52 THz.

References

see main manuscript
